# Thousands of articles on neurosurgery, yet few self-assessments of neurosurgical evidence: the need to integrate meta-research for a more reliable clinical practice

**DOI:** 10.1590/1806-9282.20251867

**Published:** 2026-05-11

**Authors:** Ivan David Lozada-Martinez, Luis Miguel Delgado Arias, Jesús Eduardo Marulanda López

**Affiliations:** 1 Universidad de la Costa, Department of Health Sciences, Biomedical Scientometrics and Evidence-Based Research Unit – Barranquilla, Colombia.; 2 Clínica Colsanitas S.A., Clínica Iberoamérica, Barranquilla – Colombia.; 3 Facultad de Ciencias para la Salud, Universidad de Manizales, Manizales, Colombia.; 4 Herseen Lab – Manizales, Colombia.

The scientific output in neurosurgery has grown consistently over the last two decades^1^, covering almost every procedure and subspecialty^1^. However, quantity does not always translate into the type of evidence needed to guide practice. A key challenge for the field is determining whether the available research addresses the most relevant clinical and methodological questions^2^. From a meta-research perspective, it is essential to identify areas where evidence is missing^3^, since these blind spots limit the progress of evidence-based neurosurgery.

Despite more than 120,000 neurosurgical publications indexed globally over the past two decades, meta-research on how this evidence is produced, reported, and validated remains nearly absent^4^. In light of this finding, we ask: given the notable growth of neurosurgical research in recent years, has there been a corresponding interest in investigating research within neurosurgery itself to determine its validity, reliability, and reproducibility?

To address this question, we implemented a structured search strategy using MeSH terms (MeSH Unique IDs: D009493; D019635; and D000098346) across PubMed Central and Scopus. The process was designed to be transparent and reproducible, combining controlled vocabulary with Boolean operators to maximize sensitivity and specificity. Given the exploratory nature of the analysis aimed at characterizing all available evidence, any document related to the meta-research approach in neurosurgery and neurosurgical procedures was included, with no exclusion criteria applied.

For example, in Scopus the search strategy was: [TITLE-ABS-KEY(Neurosurger*) OR TITLE-ABS-KEY(“Neurosurgical Procedur*”) OR TITLE-ABS-KEY(“Neurologic Surgical Procedure*”)] AND [TITLE-ABS-KEY(Meta-Research) OR TITLE-ABS-KEY(“Meta Research”) OR TITLE-ABS-KEY(Meta-science) OR TITLE-ABS-KEY(“Meta science”)]. The tags were then modified, maintaining equivalence, for use in PubMed.

Metadata were exported for bibliometric analysis. In addition, a complementary examination of co-authorship was conducted, with country-level classification by geographic region and income group according to The World Bank Country and Lending Groups Classification^5^, in order to contextualize the scope and diversity of global contributions.

The search retrieved only three relevant publications: one in Systematic Reviews (2024)^6^ and two in World Neurosurgery (both in 2022)^7,8^. Despite the thousands of neurosurgical papers indexed annually, only this small set matched the criteria, underscoring the narrow evidence base available. For comparison, a broader query on neurosurgery and neurosurgical procedures returned 123,245 documents (D009493 OR D019635), which means that meta-research evidence in neurosurgery represents 0.0024% (≈ 2.43 per 100,000 papers) of the total literature.

Analysis of co-authorship revealed contributions from four countries: the United States (n=2; 40% of all authorships), Australia (n=1; 20%), Canada (n=1; 20%), and Kenya (n=1; 20%). By region, North America accounted for 60% of the authorships, East Asia and Pacific for 20%, and Sub-Saharan Africa for 20%. In terms of income group classification, 80% of contributions came from high-income economies and only 20% from a lower-middle-income economy. The co-authorship network was limited, without consistent international clusters, and the methodological designs across the three studies were heterogeneous, preventing direct comparison or synthesis.

When considered together, the three studies illustrate the presence of different but complementary gaps in neurosurgical research. The methodological gap is clear: each publication focuses on a very specific dimension, but none of them provides a comprehensive or standardized framework. For example, one study published explored meta-research questions in the broader context of global surgery^6^; another, examined how language and geography shape the visibility of neurosurgical literature^7^; while the last publication assessed the adoption of reporting guidelines such as CONSORT and EQUATOR across journals^8^.

Although all three touch on issues of quality and equity, their approaches are fragmented, which limits comparability and reproducibility. The empirical gap is even more striking: out of more than 123,000 papers related to neurosurgery and neurosurgical procedures indexed in Scopus, only three engage directly with a meta-research perspective. In practical terms, this means that less than one in every 40,000 publications addresses how neurosurgery produces, reports, or communicates its own evidence base.

Finally, there is a knowledge gap: because these studies do not build on one another, they have not generated cumulative insights that could influence research policy, guide editorial practices, or inform the training of neurosurgeons in evidence appraisal. In other words, the field continues to produce a vast quantity of clinical data, but lacks the reflective layer that would allow it to evaluate its own practices and ensure that research truly serves patients and health systems^9,10^.

In the current era, where evidence-based medicine, meta-science, and implementation research are gaining prominence, overlooking these blind spots reduces the value and applicability of research^9,10^.

How can this gap be reduced? Journals and funding agencies should actively encourage studies that focus on neglected but clinically meaningful questions. Professional societies and research groups could promote multicenter collaborations based on standardized designs, ensuring reproducibility and comparability of results. Meta-research approaches should be integrated into neurosurgical research planning, so that gaps can be detected early.

Finally, integrating meta-research concepts into residency and fellowship programs may help build methodological literacy and develop neurosurgeons capable of scrutinizing the quality, reproducibility, and bias structure of the literature they rely upon. Such curricular integration would strengthen evidence appraisal skills and support a culture of reflective, self-correcting science within neurosurgery.

Given that only three publications were identified, a quantitative synthesis or comparative statistical analysis was not feasible, as the evidence base was insufficient to support meta-analytic aggregation or formal inferential contrasts. For this reason, a descriptive approach was adopted to allow transparent characterization of the landscape while acknowledging the scarcity of meta-research output in neurosurgery.

In conclusion, this analysis uncovered a paradox: in a discipline that publishes thousands of papers each year, only three studies addressed critical questions related to methods, reporting, evaluation, reproducibility, and incentives in neurosurgical research. This situation reflects an urgent need to recognize and close methodological, empirical, and knowledge gaps to improve internal and external validity in neurosurgery. A logical next step would be the establishment of a global or regional Neurosurgical Meta-Research Consortium, enabling coordinated collaboration, methodological standardization, and shared evaluation frameworks to advance neurosurgical science from quantity to quality.

## Data Availability

The datasets generated and/or analyzed during the current study are available from the corresponding author upon reasonable request.
